# D-histidine combated biofilm formation and enhanced the effect of amikacin against *Pseudomonas aeruginosa* in vitro

**DOI:** 10.1007/s00203-024-03918-4

**Published:** 2024-03-11

**Authors:** Haichuan Zhang, Zhongwen Mi, Junmin Wang, Jing Zhang

**Affiliations:** 1 College & Hospital of Stomatology, Anhui Medical University, Key Lab. of Oral Diseases Research of Anhui Province, Hefei, 230032 China; 2 Anhui Medical University, Hefei, Anhui 230032 China

**Keywords:** *Pseudomonas aeruginosa*, Biofilm, D-amino acid, D-histidine, Quorum sensing

## Abstract

*Pseudomonas aeruginosa* is an opportunistic gram-negative pathogenic microorganism that poses a significant challenge in clinical treatment. Antibiotics exhibit limited efficacy against mature biofilm, culminating in an increase in the number of antibiotic-resistant strains. Therefore, novel strategies are essential to enhance the effectiveness of antibiotics against *Pseudomonas aeruginosa* biofilms. D-histidine has been previously identified as a prospective anti-biofilm agent. However, limited attention has been directed towards its impact on *Pseudomonas aeruginosa*. Therefore, this study was undertaken to explore the effect of D-histidine on *Pseudomonas aeruginosa* in vitro. Our results demonstrated that D-histidine downregulated the mRNA expression of virulence and quorum sensing (QS)-associated genes in *Pseudomonas aeruginosa* PAO1 without affecting bacterial growth. Swarming and swimming motility tests revealed that D-histidine significantly reduced the motility and pathogenicity of PAO1. Moreover, crystal violet staining and confocal laser scanning microscopy demonstrated that D-histidine inhibited biofilm formation and triggered the disassembly of mature biofilms. Notably, D-histidine increased the susceptibility of PAO1 to amikacin compared to that in the amikacin-alone group. These findings underscore the efficacy of D-histidine in combating *Pseudomonas aeruginosa* by reducing biofilm formation and increasing biofilm disassembly. Moreover, the combination of amikacin and D-histidine induced a synergistic effect against *Pseudomonas aeruginosa* biofilms, suggesting the potential utility of D-histidine as a preventive strategy against biofilm-associated infections caused by *Pseudomonas aeruginosa*.

## Introduction

*Pseudomonas aeruginosa* is a prevalent human opportunistic pathogen that often induces chronic infections, ultimately leading to clinical treatment failure. *P. aeruginosa* is characterized by the ability to easily develop drug resistance, accompanied by a rapid variation in its resistance profile (Pan et al. 2022). Biofilm formation represents a critical mechanism underlying antibiotic resistance in *P. aeruginosa* infections (Ciofu et al. 2022). Once established, biofilms protect embedded bacteria from environmental stresses, immune responses, and bactericidal agents, contributing to worsened wound conditions and significant patient mortality (Moser et al. 2021). Therefore, anti-biofilm strategies and alternatives to antibiotics are essential to enhance the eradication of *P. aeruginosa* and combat associated infections.

D-amino acids are an integral part of bacterial peptidoglycans, serving as key modulators of bacterial growth and persistence (Cava et al. 2011). Studies have demonstrated the inhibitory effect of a mixture of D-amino acids (D-leucine, D-methionine, D-tyrosine, and D-tryptophan) produced by *Bacillus subtilis* during the dispersion phase, rapidly accumulating and inhibiting biofilm formation (Kolodkin-Gal et al. 2010). A mixture of D-amino acids containing D-histidine has also been demonstrated to disrupt the cell membrane of *Porphyromonas gingivalis* (Zhang et al. 2021). The activation of D-amino acids may be associated with the inhibition of extracellular protein synthesis in biofilms and the thinning of the bacterial extracellular matrix structure (Leiman et al. 2013). Furthermore, D-amino acids can inhibit the initial adhesion of bacteria by reducing hydrogen bonding and altering surface potential and hydrophilic properties (Wang et al. 2018). Moreover, numerous recent studies have demonstrated that D-amino acids inhibit biofilms formed by various pathogenic bacteria, including *P. aeruginosa*, *Staphylococcus aureus*, *Campylobacter jejuni*, and *Acinetobacter baumannii* (Elgamoudi et al. 2020; Rumbo et al. 2016a; Hochbaum et al. 2011). Therefore, D-amino acids have garnered considerable attention as novel biofilm inhibitors with multiple targets and a broad spectrum of action against drug-resistant infections. However, most studies have focused on D-tyrosine, D-tryptophan, and mixtures of D-amino acids, with limited investigations into the effects of D-histidine on *P. aeruginosa* (Jia et al. 2017; Miyamoto et al. 2020).

Quorum sensing (QS) system plays a pivotal role in biofilm maturation and bacterial responses by regulating the expression of virulence factors in a cell density-dependent manner. Therefore, this system augments bacterial virulence and determines the aggressiveness of pseudomonal infections. Previous studies have indicated the critical role of two QS systems (*Las* and *Rhl*) as key components in *P. aeruginosa* biofilm communication, offering potential targets for inhibiting developmental processes such as initial attachment, microcolony formation, and maturity (Chadha et al. 2022). The virulence of *P. aeruginosa* is strongly regulated by QS. Biofilms with a dense extracellular matrix protect bacteria, facilitating their evasion of the host immune system and the subsequent decrease in the effects of antibiotics (Moradali et al. 2017). Therefore, there is an urgent need to develop dispersal agents to disrupt the biofilm microarchitecture and improve the efficacy of antibiotics.

In this study, we aimed to evaluate the anti-biofilm effects of D-histidine against *P. aeruginosa* using quantitative real-time PCR (qRT-PCR), anti-QS activity, biofilm formation assay, and minimum inhibitory concentration (MIC) testing. The results of this study are poised to highlight the potential utility of D-histidine as a therapeutic agent against *P. aeruginosa*-associated infections.

## Materials and methods

### Chemicals and materials

D-histidine, L-histidine, azocasein, propidium iodide (PI), and fluorescein isothiocyanate-concanavalin A (FITC-ConA) were purchased from Sigma-Aldrich (St. Louis, MO, USA). DNA polymerase PrimeScrip™ RT Reagent Kit and TB Green® Premix Ex Taq™ II were purchased from TaKaRa (Dalian, China), and the RNeasy MinElute Cleanup kit was procured from QIAGEN (Hilden, Germany). These chemicals and materials were used according to the manufacturers’ instructions. Other chemicals and reagents were of analytical grade and purchased from commercial sources unless specified otherwise.

### Bacterial growth

Overnight cultures of *P. aeruginosa* were diluted to an optical density at 600 nm (OD_600_) of 0.1 in lysogeny broth (LB) medium, following a previously described protocol (Wang et al. 2022a). Subsequently, the bacterial suspension was added to 100 mM D-histidine or L-histidine and shaken. An equal volume of sterile ultrapure water was used in the control group. The turbidity of the culture was measured at 600 nm at 2-h intervals.

### QS system gene analysis

Overnight cultures of *P. aeruginosa* were diluted to an OD_600_ of 0.1 in LB medium in a 12-well cell culture plate with 0mM/100nM/10µM/1mM/50mM/100mM D-histidine. After incubating under static conditions for 24 h at 37 ℃, the bacterial cells were harvested through centrifugation and immediately subjected to RNA extraction using the RNeasy MinElute Cleanup Kit. Real-time PCR and qPCR were performed separately using the PrimeScript™ RT reagent kit. The qPCR primers are listed in Table 1. RT-PCR was performed using the LightCycler® 96 Instrument, and cycle conditions were set at 95 ℃ for 30 s, followed by 40 cycles of denaturation at 95 ℃ for 5 s and extension at 60 ℃ for 30 s. A negative control containing RNase-free water instead of cDNA was included in each run. We used the 2^−ΔΔCt^ method to calculate the relative expression of the target gene against the internal reference gene (Livak and Schmittgen 2001), where ΔCt denotes the difference between the Ct value of the target gene minus that of the internal reference gene, and ΔΔCt denotes the difference between ΔCt values across different samples.

**Table 1:** The qPCR primers for *lasI/R* and *rhlI*/*R*.

Gene	Primers (5’ − 3’)
*lasI*	F: CTACAGCCIGCAGAACGACA
	R: ACGCTCAAGTGGAAAATTGG
*lasR*	F: ATCTGGGICTTGGCATTGAG
*rhlI* *rhlR*	R: GTAGATGGACGGTTCCCAGA F: CTCTCTGAATCGCTGGAAGG R: GACGTCCTTGAGCAGGTAGG F: AGGAATGACGGAGGCTTTTT R: CCCGTAGTTCTGCATCTGGT
*16s rRNA*	F: GTGGTTCAGCAAGTTGGA
	R: CCTCAGTGTCAGTATCAGTC

### QS-regulated pyocyanin, alginate, and proteolytic activity

Overnight cultures of *P. aeruginosa* were diluted to an OD_600_ of 0.1 in LB medium in a 12-well cell culture plate with 0mM/100nM/10µM/1mM/50mM/100mM D-histidine or L-histidine. After incubating under static conditions for 24 h at 37 ℃, cultures for the determination of virulence factors. Pyocyanin content was determined according to a previously reported method (Wang et al. 2022b), with slight modifications. The supernatant, obtained after centrifugation at 8000 rpm, was mixed with an equal volume of trichloromethane and centrifuged. Organic solvents were extracted with equal volumes of 0.2 M HCl. The absorbance of the extract was measured at 520 nm using a microplate reader. The change in pyocyanin yield was calculated based on the percentage change in absorbance. Alginate content was measured according to previously described methods (Yasuda et al. 1993), with slight modifications. The bacterial suspension (1 mL) was mixed with 3 mL of a 10% CuSO_4_ solution. The reaction mixture was adjusted to a pH of 4.0 using 1 M HCl, maintained at 25 ℃ for 1 h, and centrifuged at 13,000 rpm for 2 min. The precipitate was redissolved in 0.1 mL of 1 M NH_4_OH and diluted with 0.9 mL of water. The sample (1 mL) was treated with 2 mL of copper-HCl reagent and 1 mL of naphthoresorcinol reagent and placed in a boiling water bath for 40 min. After allowing it to cool, 4 mL of butyl acetate was added to the mixture, shaken thoroughly, and centrifuged to separate the butyl acetate layers. After washing once with 20% NaCl solution, the optical density at 565 nm was measured. The change in alginate yield was calculated based on the percentage change in absorbance. Proteolytic activity was measured using azocasein as the substrate according to a previously described method (Ayora and Götz 1994), with slight modifications. Briefly, 200 µL of cell-free supernatant (both treated and untreated) was added to 50 µL of 0.3% azocasein in 0.05 M Tris–HCl (pH 7.5). The reaction mixture was subsequently incubated at 37 ℃ for 60 min and stopped by adding l0% trichloroacetic acid. The absorbance of the clear supernatant was measured at 400 nm. The change in proteolytic yield was calculated based on the percentage change in absorbance.

### Swarming and swimming motility assays

Motility was determined in two types of media, following a previously described method (Wang et al. 2014), with slight modifications. The medium used for the swarming motility assay comprised 0.5% agar, 0.8% nutrient broth, and 0.5% glucose with or without D-histidine or L-histidine. The medium used for the swimming motility assay comprised 0.3% agar, 0.5% NaCl, and 1% peptone, with or without D-histidine or L-histidine. All plates were solidified overnight at 25 ℃ before use. Overnight cultures were spotted onto swarming or swimming agar plates using a toothpick. After incubation at 37 ℃ for 24 h, the swarming and swimming motility diameters were measured.

### Biofilm formation

Following a previous study (Wang et al. 2022a), we used the wild-type *P. aeruginosa* PAO1 strain, as preserved in our laboratory, in this study. PAO1 was grown on LB agar plates at 37 ℃ overnight. Monoclonal colonies were scraped from the plates, inoculated into fresh LB medium, and incubated for 8 h with shaking. Subsequently, cultures of *P. aeruginosa* were diluted to an OD_600_ of 0.1 in LB medium in either 96-well plates or NEST glass-bottom cell culture dishes at 0mM/100nM/10µM/1mM/50mM/100mM D-histidine or L-histidine. An equal volume of sterile, ultrapure water was added to the control group. After 12 h of cultivation at 37 ℃, planktonic cells were aspirated, and the plates were mildly washed thrice with phosphate-buffered saline (PBS). Biofilm formation was measured using crystal violet staining or confocal laser scanning microscopy (CLSM).

For the crystal violet assay, each biofilm well was stained with 0.1% crystal violet solution for 30 min. Subsequently, the microplates were washed thrice with PBS and dried for 20 min, after which biofilm formation was quantified by measuring absorbance at 590 nm following solubilization in 95% ethanol.

For confocal imaging, the cells were stained with PI for 15 min in the dark at 25 ℃ and washed thrice with PBS. Next, the extracellular polysaccharide components of the biofilms were stained with FITC-ConA for 15 min in the dark at 25 ℃, followed by three sessions of washing with PBS. Cells and biofilms were fixed with 2.5% glutaraldehyde. After perfusion fixation, each plate was stored using 60% glycerin at 4 ℃. The stained plates were observed and photographed using a laser confocal microscope.

### Dispersal assay

Overnight cultures of *P. aeruginosa* were diluted to an OD_600_ of 0.1 in an LB medium placed in a 12-well cell culture plate, following a previously described method (Wang et al. 2022a). After incubation under static conditions for 72 h at 37 ℃, planktonic cells were aspirated, and the plates were washed mildly thrice with PBS. Next, 0mM/100nM/10µM/1mM/50mM/100mM D-histidine or L-histidine were added to the cells, followed by incubation for 24 h. The methods described above were used to detect remaining biofilms.

### Minimum inhibitory concentration

Minimum inhibitory concentration (MIC) was conducted following a previously reported protocol (Behera et al. 2019; Zhang et al. 2023), with slight modifications. Antibiotic susceptibility was determined based on MIC using the microdilution method. Briefly, a 96-well microplate containing two-fold serial dilutions of the antibiotic amikacin, with concentrations ranging from 25.6 µg/mL to 0.05 µg/mL, was prepared. Media containing 0mM/100nM/10µM/1mM/50mM/100mM D-histidine or L-histidine were added to the experimental groups. An equal volume of sterile, ultrapure water was added to the control group. Overnight cultures in LB were diluted in fresh LB medium to an OD_600_ of 0.1. Next, 100 µL of the culture solution was dispensed into 96-well microplates. MIC was measured at 600 nm after 12 h of incubation.

### Statistical analysis

Statistical analyses were performed using GraphPad Prism version 9.5.0. Data are expressed as the means ± standard deviation. Dunnett’s t-test was used to compare the test and control groups. Data analysis was performed using SPSS version 24.0. Values of *P* < 0.05 were considered statistically significant. All experiments were performed in triplicate and repeated thrice.

## Results

### Effect of D-histidine on the growth of ***P. aeruginosa*** PAO1

**Fig. 1:**
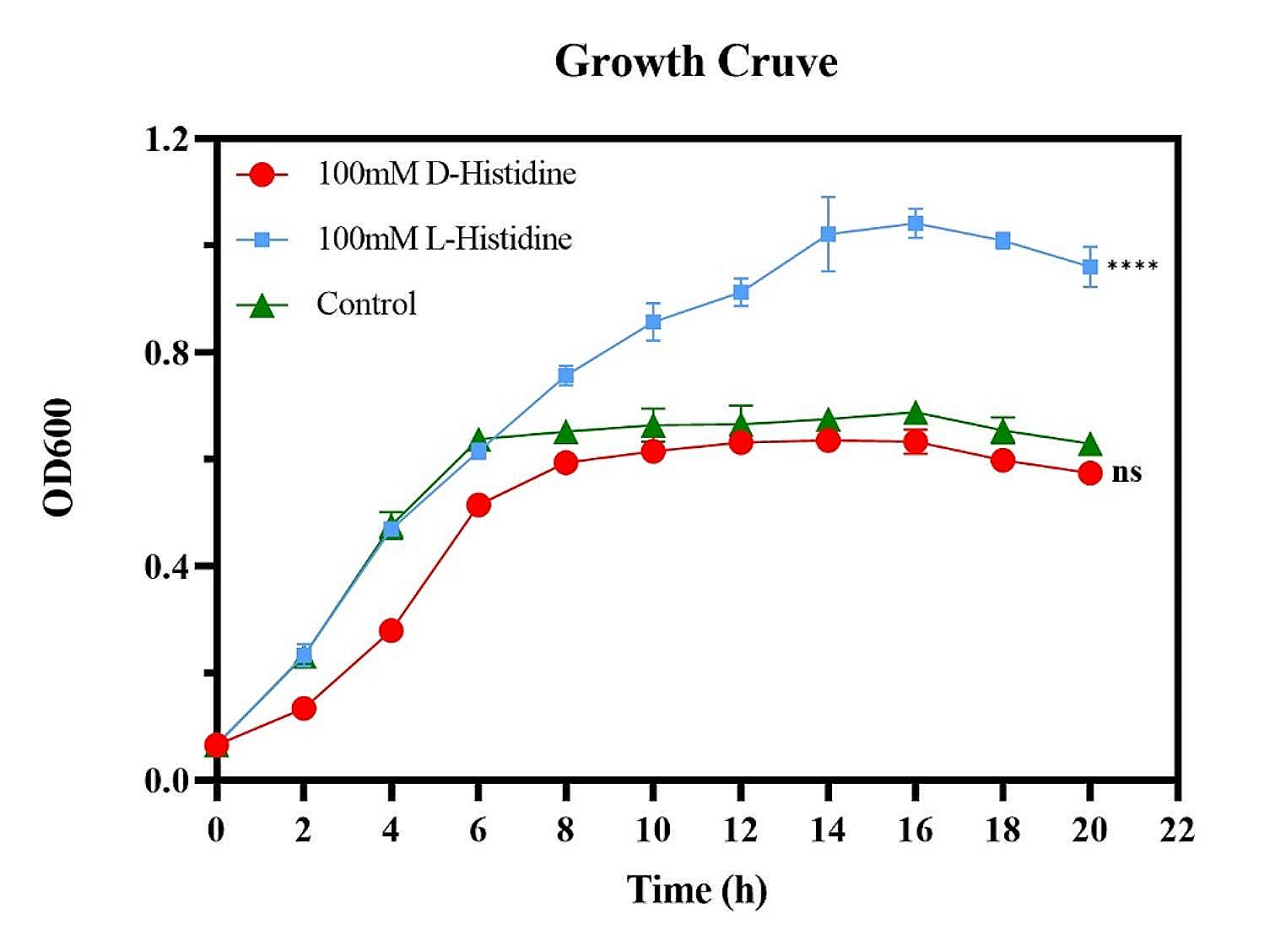
Effect of D-histidine and L-histidine on the growth of *P. aeruginosa* PAO1. PAO1 cells grown overnight were treated with 100 mM D-histidine and L-histidine, and every 2 h cells were carefully transferred to another microtiter plate for total 20 h, and OD_600_ nm was read. The error bars represent standard errors. ns, not significant, **** *P* < 0.0001 (vs. control)

We generated growth curves (Fig. 1) to determine the effect of D-histidine on the growth of *P. aeruginosa* PAO1. The growth curves indicated that D-histidine, at 100 mM, exerted no significant inhibitory effect on the growth of *P. aeruginosa* PAO1. Subsequent investigations involved using 100 mM and lower concentrations of D-histidine to explore the possible effects of D-histidine on the QS system, virulence factors, and biofilm formation of *P. aeruginosa* PAO1.

### Effect of D-histidine on the expression of QS system genes

**Fig. 2:**
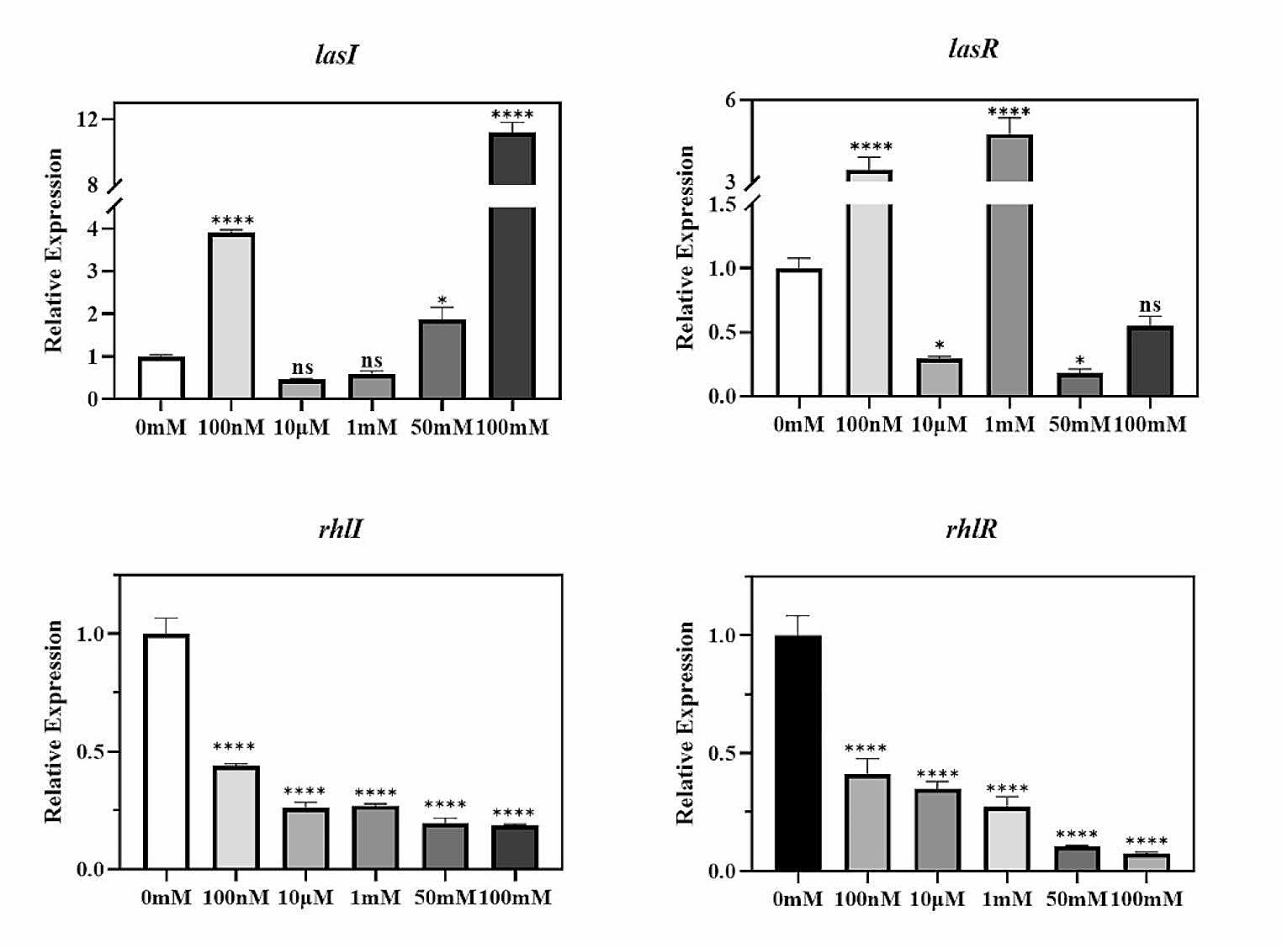
Effect of D-histidine at concentrations ranging from 100 nM to 100 mM on the expression of *P. aeruginosa* PAO1 QS genes. The ordinate represents relative expression. The error bars represent standard errors. ns, not significant, * *P* < 0.05, **** *P* < 0.0001 (vs. control)

The *Las* and *Rhl* systems constitute the most widely used QS systems. RT-qPCR analysis was conducted to elucidate the potential influence of D-histidine on the QS system and identify the specific pathway it might disrupt. The results (Fig. 2) revealed no regularity in the regulation of the *Las* system with varying concentrations of D-histidine; conversely, a reduction in the expression of both *Rhl*I and *Rhl*R genes was observed. At a concentration of 100 mM, the expression of *Rhl*I and *Rhl*R genes was downregulated by approximately 80% and 90%, respectively (Fig. 2). *Rhl*I synthesizes C4-HSL, which, in conjunction with its cognate receptor *Rhl*R, drives the expression of virulence genes involved in pyocyanin production and the regulation of bacterial motility (Papenfort and Bassler 2016). In the subsequent experiments, we explored the effects of D-histidine on QS-regulated virulence factors.

### Effect of D-histidine on QS-regulated pyocyanin, alginate, and proteolytic activity

**Fig. 3:**
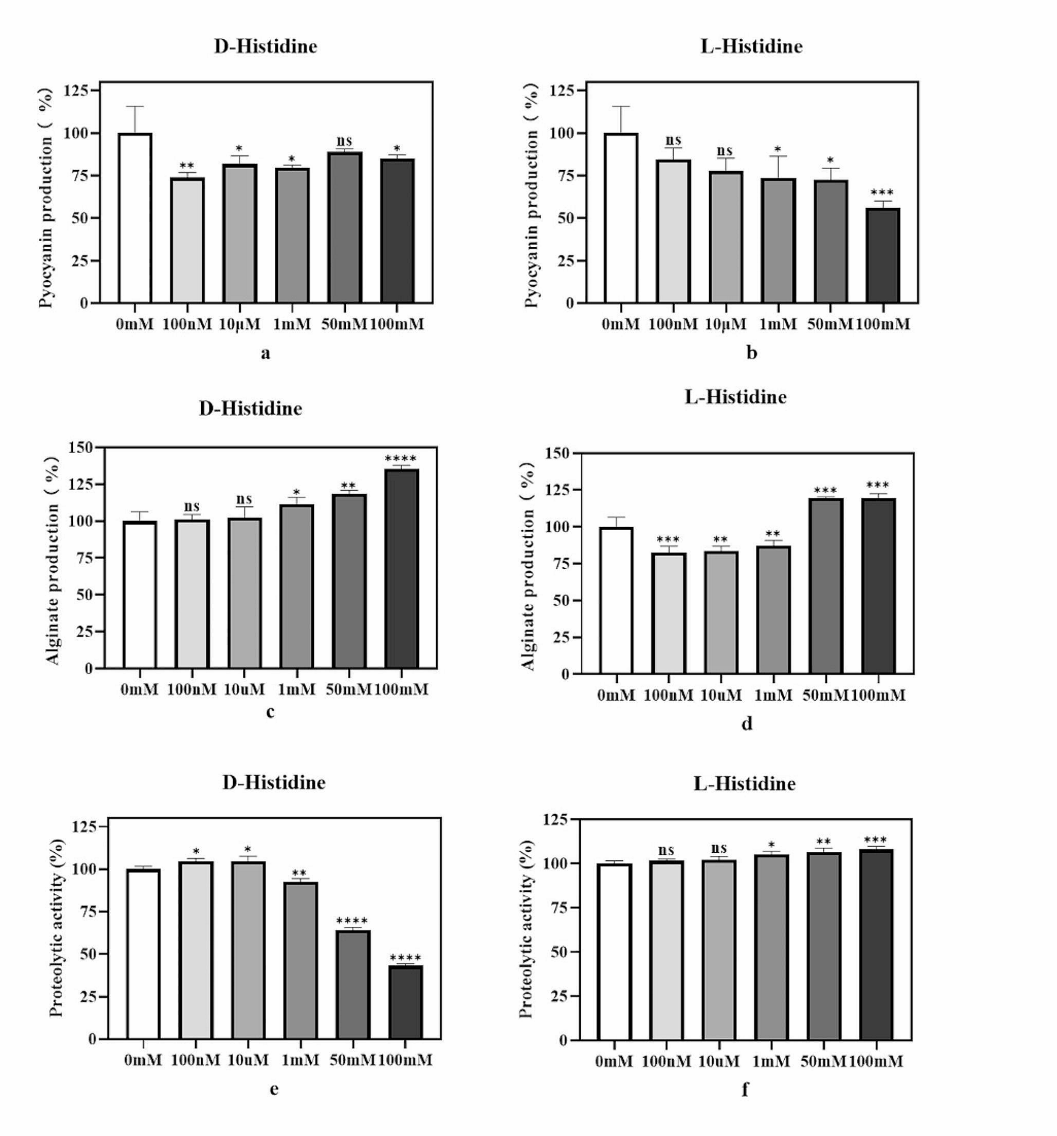
Effect of D-histidine and L-histidine at concentrations ranging from 100 nM to 100 mM on the QS-regulated virulence factors. Pyocyanin **(a, b)**, alginate **(c, d)** and proteolytic activity **(e, f)** were assayed in the presence and absence of the D-histidine and L-histidine. The ordinate represents relative expression, the change in pyocyanin, alginate and proteolytic yield were calculated based on the percentage change in absorbance. The error bars represent standard errors. ns, not significant, * *P* < 0.05, ** *P* < 0.01, *** *P* < 0.001, ****, *P* < 0.0001 (vs. control)

We assessed the impact of D-histidine on three typical virulence factors regulated by QS in *P. aeruginosa*. Pyocyanin decreased irregularly at varying D-histidine concentrations, and a relatively weak inhibitory effect was observed (Fig. 3a). However, D-histidine increased alginate secretion, particularly at high concentrations (Fig. 3c). Conversely, high concentrations of D-histidine inhibited proteolytic activity, whereas low concentrations of D-histidine did not. At 100 mM, proteolytic activity decreased by approximately 57% (Fig. 3e).

### Effect of D-histidine on the motility of ***P. aeruginosa***

**Fig. 4:**
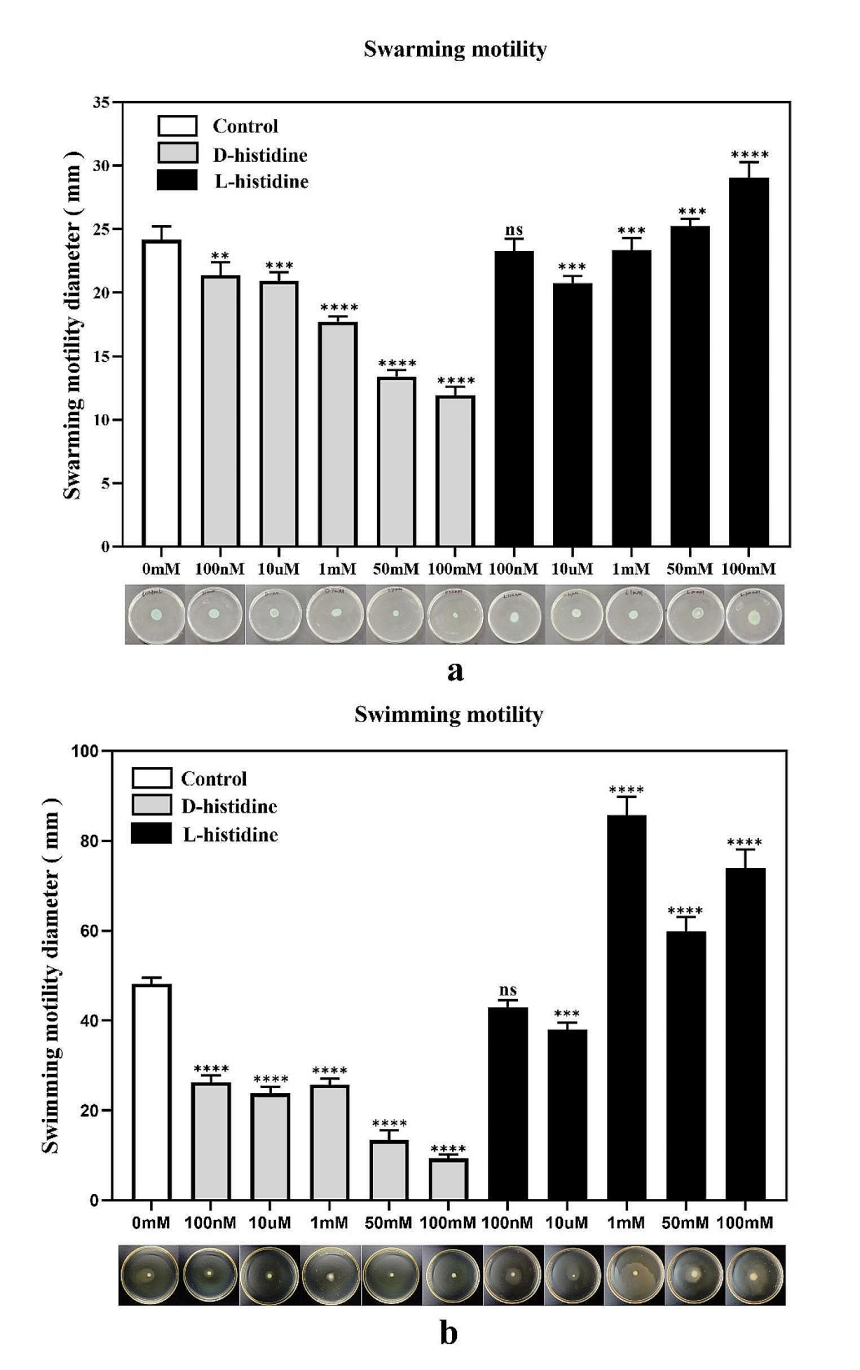
Effect of D-histidine and L-histidine at concentrations ranging from 100 nM to 100 mM on the swarming motility **(a)** and swimming motility **(b)** of *P. aeruginosa* PAO1. Upper panel: Graphs depicting the diameters of bacterial growth. Lower panel: Growth of bacteria on motility plates containing different concentrations of D-histidine and L-histidine. The error bars represent standard errors. ns, not significant, ** *P* < 0.01, *** *P* < 0.001, **** *P* < 0.0001 (vs. control)

High concentrations of D-histidine inhibited *P. aeruginosa* swarming and swimming motility in a dose-dependent manner (Fig. 4). At a concentration of 100 mM, D-histidine decreased the rates of swarming and swimming motility to 32% and 19%, respectively. In contrast, high concentrations of L-histidine promoted *P. aeruginosa* swarming and swimming motility.

### Effect of D-histidine on ***P. aeruginosa*** biofilm formation

**Fig. 5:**
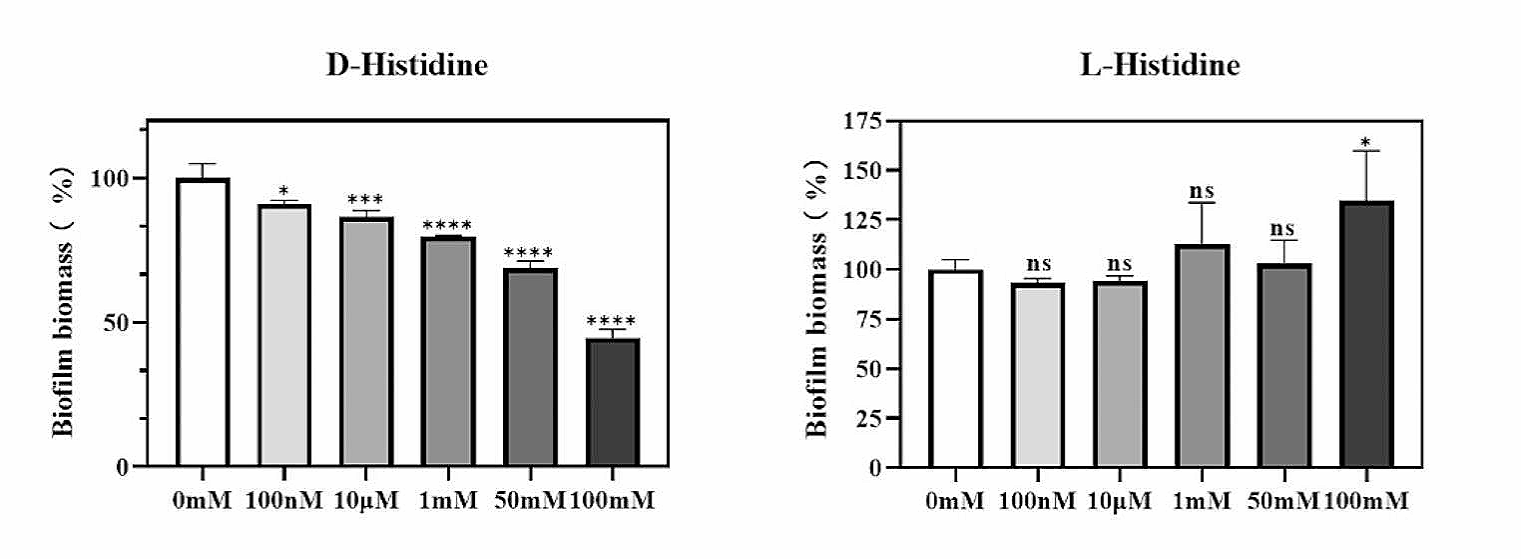
Effect of D-histidine and L-histidine at concentrations ranging from 100 nM to 100 mM on biofilm formation by *P. aeruginosa* PAO1. The biofilm formation was determined by crystal violet assay. The change in biofilm biomass was calculated based on the percentage change in absorbance. The error bars represent standard errors. ns, not significant, * *P* < 0.05, *** *P* < 0.001, **** *P* < 0.0001 (vs. control)

**Fig. 6:**
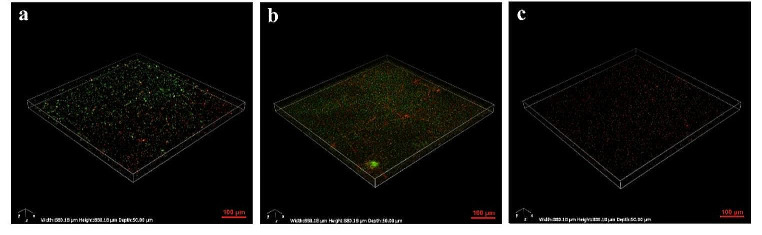
Confocal laser scanning microscopy (CLSM) images of biofilm formation on NEST glass-bottom cell culture dishes. Control group **(a)**, cultures with 100 mM L-histidine **(b)** and cultures with 100 mM D-histidine **(c)**

The assessment of the impact of D-histidine on *P. aeruginosa* biofilm formation involved a two-step approach. Initially, a crystal violet quantitative biofilm assay was used to detect the inhibitory effect of D-histidine at varying concentrations. After the optimal concentration of D-histidine was determined, CLSM was used to assess the biofilm inhibition effect. The crystal violet staining assay demonstrated the inhibitory effect of D-histidine on *P. aeruginosa* PAO1 biofilm formation (Fig. 5). Moreover, as D-histidine concentration increased, this effect became more evident. The most significant effect was observed at an D-histidine concentration of 100 mM, resulting in a 55% inhibition of biofilm formation. This result was consistent with that of CLSM (Fig. 6). In contrast, opposite results were observed for L-histidine (Fig. 5), where biofilm volume became increasingly evident with higher concentrations of L-histidine. At a concentration of 100 mM L-histidine, biofilm volume was enhanced by 1.35-fold compared to that of the control group.

### Effect of D-histidine on mature biofilm of ***P. aeruginosa***

**Fig. 7:**
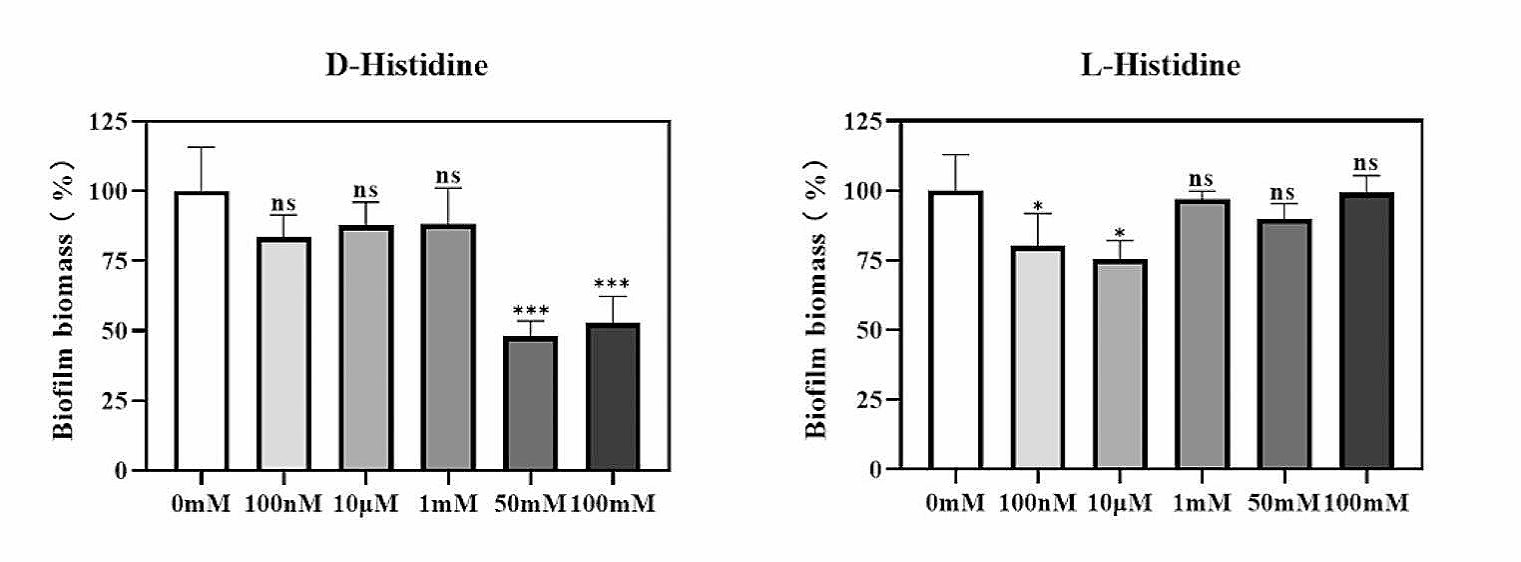
Effect of D-histidine and L-histidine at concentrations ranging from 100 nM to 100 mM on mature biofilm of *P. aeruginosa* PAO1. 72 h mature biofilms were cultured overnight with D-histidine and L-histidine and the quantification of biofilm formation determined by crystal violet assay. The change in biofilm biomass was calculated based on the percentage change in absorbance. The error bars represent standard errors. ns, not significant, * *P* < 0.05, *** *P* < 0.001 (vs. control)

Similar to the method employed for the biofilm formation assay, the biofilm dispersing effect was quantified using crystal violet staining. Each concentration of D-histidine demonstrated the ability to disperse the formed biofilm, specifically at high concentrations (Fig. 7). In the presence of 100 mM/50 mM D-histidine, biofilm dispersion reached approximately 50%. Compared to that observed for D-histidine, the biofilm exhibited minimal changes after L-histidine intervention.

### Effect of D-histidine on the antibacterial activity of amikacin

**Fig. 8:**
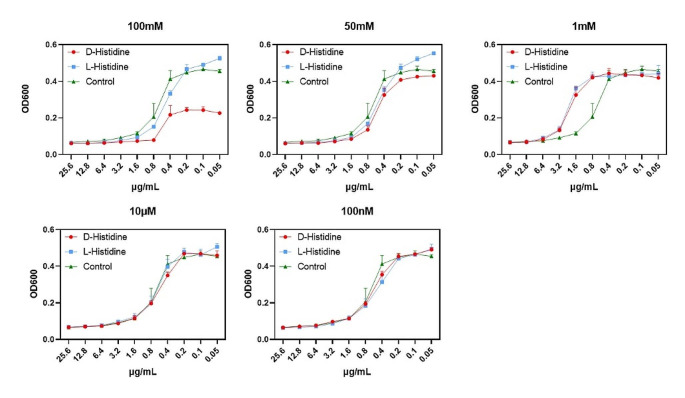
Results of minimum inhibitory concentrations for *P. aeruginosa* PAO1. There exists a region of antibiotics concentrations within ranges of 0.05–25.60 µg/mL. The experimental groups were exposed to D-histidine and L-histidine solutions at concentrations ranging from 100 nM to 100 mM. The same volume of sterile ultrapure water was added to the control group. The error bars represent standard errors

To analyze the effect of D-histidine on antibiotic sensitivity, we examined the MIC of amikacin and compared it with that of L-histidine. The results revealed that D-histidine increased antibiotic sensitivity to amikacin, with a more significant effect compared to that of L-histidine. The MIC of control amikacin was 1.6 µg/mL (Fig. 8). However, the addition of 100 mM D-histidine decreased the MIC of amikacin to 0.8 µg/mL. In contrast, L-histidine did not exhibit a similar reduction in the MIC of amikacin. These results suggest that D-histidine exhibited a synergistic effect with antibiotic treatment and may promote the effect of antibiotics.

## Discussion

Biofilm formation is the initiating factor in *P. aeruginosa* infections, which subsequently induce acute and chronic infections in patients with severe burns, metabolic diseases, and cystic fibrosis, among others (Nick et al. 2022). Antibiotics exhibit strong bactericidal efficacy against planktonic *P. aeruginosa* (Horcajada et al. 2019); however, extensive and long-term exposure to systemic antibiotics increases the risk of bacterial resistance. The presence of a dense extracellular matrix within biofilms hinders the permeation of antibiotics, facilitating the development of antibiotic-resistant bacterial strains (Lebeaux et al. 2014). Therefore, there is an urgent need for an ideal biofilm-disrupting agent with the ability to enhance the efficacy of antibiotics against *P. aeruginosa*.

D-amino acids exhibit favorable pharmacokinetic properties and a lack significant toxicity, rendering them potential candidates for preventing and treating various biofilm-associated infections (Bardaweel et al. 2013; Meftah et al. 2021). The growth curve analysis of *P. aeruginosa* treated with 100 mM D-histidine showed that D-histidine did not noticeably inhibit the bacterial activity of *P. aeruginosa*. Previous studies have demonstrated that D-tyrosine permanently prohibits biofouling (without affecting bacterial growth) by eliminating adhesion and biofilm formation of *P. aeruginosa* on nanofiltration membranes (Yu et al. 2012). However, different concentrations of a D-amino acid mixture containing D-histidine were observed to delay *P. gingivalis* proliferation without inhibiting the growth of *Streptococcus sanguinis* and *Actinomyces viscosus* (Zhang et al. 2021). This observed variation may be related to the species specificity of D-amino acids (Vahdati et al. 2022).

The virulence of *P. aeruginosa* is stringently regulated by a cell density-dependent phenomenon called QS. This phenomenon directs the social behavior of the pathogen and synchronizes biological responses at the community level to activate bacterial persistence and survival. QS plays a key role in the entire pathogenesis of *P. aeruginosa*, from host colonization to invasion, infection, dissemination, immune evasion, and drug resistance. Moreover, it enhances bacterial virulence and dictates the aggressiveness of Pseudomonas infections. Our investigation revealed that D-histidine downregulated the mRNA expression of QS-associated genes, such as *RhlI/RhlR*. A previous study reported that the *Rhl* system regulates *P. aeruginosa* pyocyanin production and bacterial motility (Papenfort and Bassler 2016). Consistent with the findings of a previous study, we observed that high concentrations of D-histidine reduced the production of *P. aeruginosa*-associated virulence factors, such as pyocyanin, as well as proteolytic activity. Moreover, *P. aeruginosa* biofilm formation is regulated by the *Las* system (Rutherford and Bassler 2012). However, the regulation of the *Las* system by D-histidine exhibited irregularities and even increased gene expression in this study. This result suggests the existence of other mechanisms of action for D-histidine, potentially influencing biofilm formation through alternative pathways.

Various D-amino acids, such as D-tryptophan, D-phenylalanine, D-tyrosine, and D-methionine, have demonstrated effective inhibition of biofilm formation and disruption of mature biofilms of *P. aeruginosa* (Brandenburg et al. 2013; Sanchez et al. 2014; She et al. 2015). The motility of pathogenic bacteria is a contributing factor to colonization and biofilm formation (Palamae et al. 2023). The results of this study revealed that high concentrations of D-histidine inhibited the swarming and swimming motility of *P. aeruginosa*. The action of D-amino acids is dose- and species-specific (Yu et al. 2016), where the intervention concentration may influence the inhibitory effect on biofilm formation. Kao WTK et al. demonstrated that D-tryptophan, at concentrations of 10 µM and 1 mM, exerted no inhibitory effect on the biofilm of *P. aeruginosa* (Kao et al. 2017), whereas concentrations of 4 mM or 5 mM exhibited biofilm inhibition activity (Rumbo et al. 2016b; Chang et al. 2022). Consistent with these findings, we confirmed that 50 mM/100 mM of D-histidine inhibited biofilm formation and induced mature biofilm disassembly, whereas low concentrations of D-histidine had no significant effect. D-amino acids may inhibit biofilm formation via diverse mechanisms. Xing et al. revealed that D-amino acids are associated with the repulsive nature of bacterial cells, inhibiting initial bacterial adhesion (Xing et al. 2015). Xu et al. indicated that D-tyrosine inhibited the production of AI-2 in a mixed bacterial culture, suggesting the potential to inhibit biofilm formation (Xu and Liu 2011). In this study, D-histidine may have affected *P. aeruginosa* biofilm by downregulating the expression of QS system-related genes and affecting bacterial motility. However, the mechanism underlying the effects of D-histidine on bacterial biofilms remains unexplored and warrants further investigation.

Amikacin, a semisynthetic analog of kanamycin, demonstrates strong activity against most gram-negative bacteria. Planktonic *P. aeruginosa* is typically susceptible to the effects of amikacin. However, mature biofilms exhibit a three-dimensional architecture that resists antibiotic permeation. The antimicrobial potency of anti-biofilms and biocides to inhibit biofilm can be significantly improved by combining them with D-amino acids (Worthington and Melander 2013). The combined effect of D-tyrosine and amikacin against *P. aeruginosa* has been previously reported. D-tyrosine improved the efficacy of amikacin in combating *P. aeruginosa* biofilms, as indicated by a reduction in the minimal biofilm-inhibiting concentrations (MBIC50 and MBIC90), without a change in the MIC of planktonic bacteria (She et al. 2015). In our study, 100 mM D-histidine exhibited synergistic effects with amikacin and reduced the MIC of amikacin. Previous studies have indicated that a mixture of D-amino acids containing D-histidine disrupts the cell membrane of *P. gingivalis*. It is plausible that D-histidine may modulate bacterial membrane stability and permeability to increase the susceptibility of *P. aeruginosa* to amikacin.

In conclusion, our findings revealed that D-histidine exerted inhibitory effects on *Rhl* system-related genes, pyocyanin production, proteolytic activity, and motility, thereby efficiently impeding the biofilm formation of *P. aeruginosa*. The MIC results further demonstrated a synergistic effect between D-histidine and the antibiotic amikacin. However, the precise mechanism of action of D-histidine remains unclear and requires further investigation. We tested the elucidation of D-histidine on PAO1 stain only in this study. It is needed to explore the effects of D-histidine on variation *P. aeruginosa* strains, including antibiotics strains. Therefore, further research is necessary to explore the possible mechanisms and specific areas for D-histidine application.

## Data Availability

Data used in the present study are available from the corresponding author on reasonable request.
